# Effect of Multiple Reflows on the Interfacial Reactions and Mechanical Properties of an Sn-0.5Cu-Al(Si) Solder and a Cu Substrate

**DOI:** 10.3390/ma14092367

**Published:** 2021-05-02

**Authors:** Junhyuk Son, Dong-Yurl Yu, Yun-Chan Kim, Shin-Il Kim, Min-Su Kim, Dongjin Byun, Junghwan Bang

**Affiliations:** 1 Micro-Joining Center, Korea Institute of Industrial Technology, 156 Gaetbeol-ro, Yeonsu-gu, Incheon 406-840, Korea; jhson@kitech.re.kr (J.S.); alpha0987@kitech.re.kr (D.-Y.Y.); kyc20@kitech.re.kr (Y.-C.K.); shinil93@kitech.re.kr (S.-I.K.); mskim927@kitech.re.kr (M.-S.K.); 2 Department of Material Science and Engineering, Korea University, Anam-dong, Seongbuk-gu, Seoul 136-713, Korea; dbyun@korea.ac.kr; 3 Department of Materials Science and Engineering, Seoul National University of Science and Technology, Seoul 01811, Korea

**Keywords:** electric control unit (ECU), lead-free solder, Al(Si), intermetallic compounds (IMCs), multiple reflow

## Abstract

In this study, the interfacial reactions and mechanical properties of solder joints after multiple reflows were observed to evaluate the applicability of the developed materials for high-temperature soldering for automotive electronic components. The microstructural changes and mechanical properties of Sn-Cu solders regarding Al(Si) addition and the number of reflows were investigated to determine their reliability under high heat and strong vibrations. Using differential scanning calorimetry, the melting points were measured to be approximately 227, 230, and 231 °C for the SC07 solder, SC-0.01Al(Si), and SC-0.03Al(Si), respectively. The cross-sectional analysis results showed that the total intermetallic compounds (IMCs) of the SC-0.03Al(Si) solder grew the least after the as-reflow, as well as after 10 reflows. Electron probe microanalysis and transmission electron microscopy revealed that the Al-Cu and Cu-Al-Sn IMCs were present inside the solders, and their amounts increased with increasing Al(Si) content. In addition, the Cu_6_Sn_5_ IMCs inside the solder became more finely distributed with increasing Al(Si) content. The Sn-0.5Cu-0.03Al(Si) solder exhibited the highest shear strength at the beginning and after 10 reflows, and ductile fracturing was observed in all three solders. This study will facilitate the future application of lead-free solders, such as an Sn-Cu-Al(Si) solder, in automotive electrical components.

## 1. Introduction

The use of Pb-bearing solders has been restricted due to international environmental regulations, such as legislation for end-of-life vehicles (ELVs) and waste electric and electronic equipment (WEEE) [1,2]. Such Pb-bearing solders have been replaced by Sn-Ag-Cu solder in electronic products, for which the usage environment is relatively less severe [3]. However, the development of more reliable soldering materials is required for automotive engine rooms and transmissions, where the temperature reaches 150 °C, and vibrations ranging from 3 G to 20 G are produced during driving. Sn-Ag-Cu, Sn-Cu, Sn-Zn, Sn-Sb, and Sn-Au solders have been considered as solder candidates for application in automobiles [4,5,6,7]. However, Sn-Ag-Cu and Sn-Ag solders have low reliability because of the Ag_3_Sn intermetallic compounds (IMCs) that affect their thermal shock resistance and their vibration life [8]. In addition, the use of Sn-Zn solder has been restricted owing to the voids and low wettability caused by the oxidation problem [9]. Sn-Sb solder can be used at high temperatures owing to its relatively high melting point, but its continuous use is unpredictable because the harmfulness of Sb has yet to be verified. The use of Sn-Au has been restricted because of the increase in the price of Au. Sn-Cu solder is inexpensive and has a high melting point and appropriate mechanical strength, but the growth of IMCs at the Sn-Cu/Cu interface occurs much faster than at the interfaces of other lead-free solders [10]. Meanwhile, solder joints are generally subjected to the reflow process more than two times due to repeated mounting and rearrangement [11,12,13]. In the reflow process, the formation of IMCs at the solder/substrate interface is essential. However, the excessive growth of IMCs may cause thermal aging and the occurrence of brittle cracks during cycling [14,15]. Therefore, research on the formation and growth of IMC layers in multiple reflows or thermal aging environments is essential [16,17]. In this regard, studies have been conducted to improve microstructures and mechanical properties by adding small amounts of trace elements to Sn-Cu solder with relatively high melting points [18,19,20,21,22]. The addition of trace elements can reduce the diffusion velocity of IMCs by lowering the activity of Cu and Su elements or by blocking the diffusion path of atoms in liquid–solid reaction conditions [23,24]. In this study, ecofriendly Sn-0.5Cu-(0.01, 0.03 wt%)Al(Si) solder compositions with a melting point of 230 °C were developed as high-reliability soldering materials, whose thermal reliability and solder joint properties were analyzed in terms of the Al(Si) content. The insides of the solders were also analyzed, and the interfacial reactions in the solder joints were observed to evaluate the applicability of the developed materials in high-temperature soldering for automotive electronic components.

## 2. Materials and Methods

**Figure 1:**
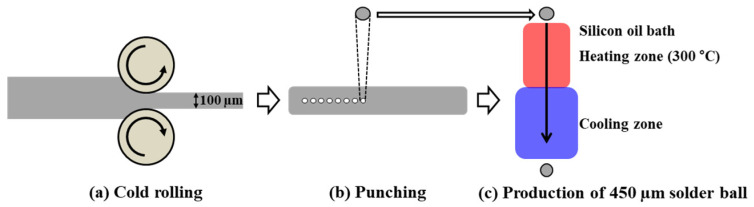
Schematic diagram of the solder ball manufacturing process.

Pure Sn (99.9%, MKE Co., Ltd., Yongin, Gyeonggi-do, Korea), Cu (99.9%, MKE Co., Ltd.), and Al(Si) alloys were used as raw materials to prepare the SC-0.01Al(Si) and SC-0.03Al(Si) solders. Instead of pure Al, a commercial Al 4047 alloy (MKE Co., Ltd.) was used due to workability and oxidation problems that could arise with pure Al during the casting process. First, pure Sn and Cu were melted in a vacuum furnace filled with argon gas at 500 °C to prepare an Sn-0.5Cu alloy, which was then cooled in air. Then, the Sn-0.5Cu alloy and Al(Si) alloy were heated to 900 °C and mixed for 20 min. After being furnace-cooled to 600 °C, the mixed alloy was kept at 600 °C for 10 min, after which it was air-cooled to 50 °C. In addition, the prepared solder alloy was homogenized at 600 °C for 1 h. The prepared solder alloy was subjected to inductively coupled plasma (ICP) analysis, the results of which are shown in Table 1. The solder balls were manufactured by punching a solder alloy after cold rolling. The punched solder alloy was immersed in a silicone oil bath system at 300 °C to form a perfectly round ball, then cooled in a rosin mildly activated (RMA)-type flux. The silicone oil bath system was used to minimize the oxidation of the solder balls during the process. The solder ball manufacturing process of the manufactured solder alloy is shown in Figure 1.

**Figure 2:**
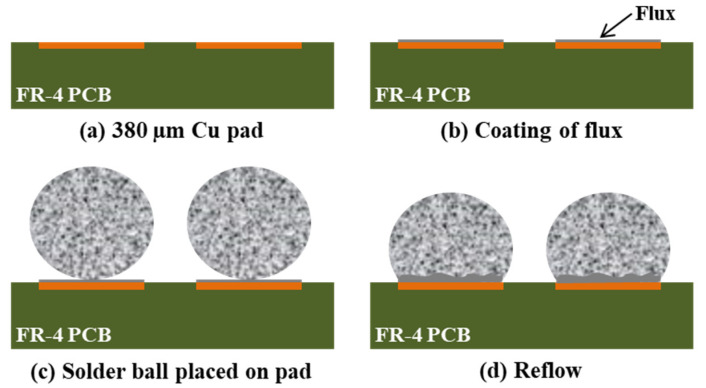
Schematic diagram of the solder ball coupon fabrication.

**Figure 3:**
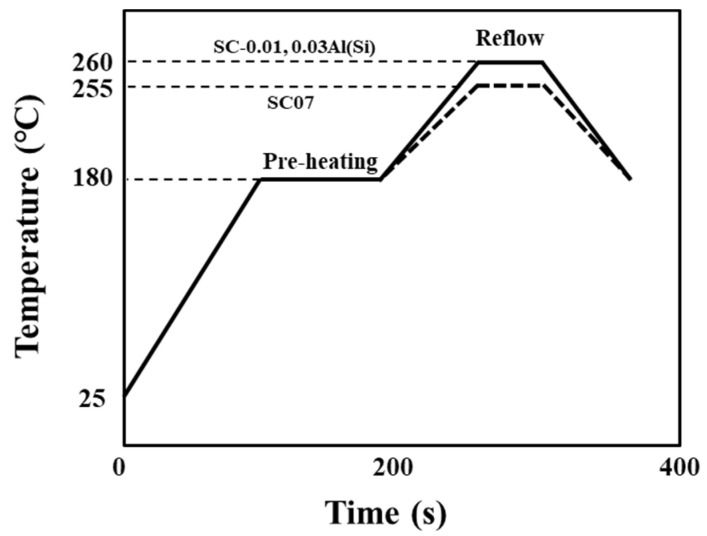
Reflow profiles of the SC07, SC-0.01Al(Si), and SC-0.03Al(Si) solders.

The melting points of the solders with the addition of Al(Si) were measured and compared using differential scanning calorimetry (DSC) analysis. After stabilizing 10 mg of the solder balls at 50 °C, their melting points were measured by increasing the temperature to 250 °C at a rate of 10 °C/min and decreasing it at a rate of 10 °C/min. To compare the changes in the solder joints during multiple reflows, a printed circuit board (PCB) flux that was treated with organic solderability preservative (OSP) Cu was applied in small quantities [25], and then the Sn-0.7Cu(SC07), Sn-0.5Cu-0.01Al(Si)(SC-0.01Al(Si)), and Sn-0.5Cu-0.01Al(Si)(SC-0.03Al(Si)) solder balls were mounted and soldered. Figure 2 shows a schematic diagram of the solder ball coupon fabrication. The solder joint properties were then evaluated by repeating the reflow process up to ten times. Figure 3 shows the reflow temperature profiles of the SC07, SC-0.01Al(Si), and SC-0.03Al(Si) solders. The Al(Si)-added solder proceeded to reflow at a temperature that was 5 °C higher than that of the SC07 solder.

The microstructures and interfacial IMCs as a result of the multiple reflows and Al(Si) addition were observed using field emission scanning electron microscopy (FE-SEM), energy dispersive spectrometry (EDS), electron probe microanalysis (EPMA), and transmission electron microscopy (TEM). After being polished with SiC paper and an Al_2_O_3_ polishing solution to observe the interfacial IMCs and the internal microstructure of the solder, it was etched with a solution of 95% C_2_H_5_OH, 3% HNO_3_, and 2% HCl (in vol.%). The following equation was used to calculate the thickness of the IMC layer [26]:

*H*_IMC_ = *H*_SEM_ × *N*_IMC_/*N*_SEM_, (1)

where *H*_SEM_ is the actual height of the SEM image and *N*_IMC_ and *N*_SEM_ are the number of pixels in the IMC layer and the entire SEM image, respectively. To increase the reliability of the thickness measurements of the IMC layer, the IMC thickness was calculated as an average of 20 SEM images.

**Figure 4:**
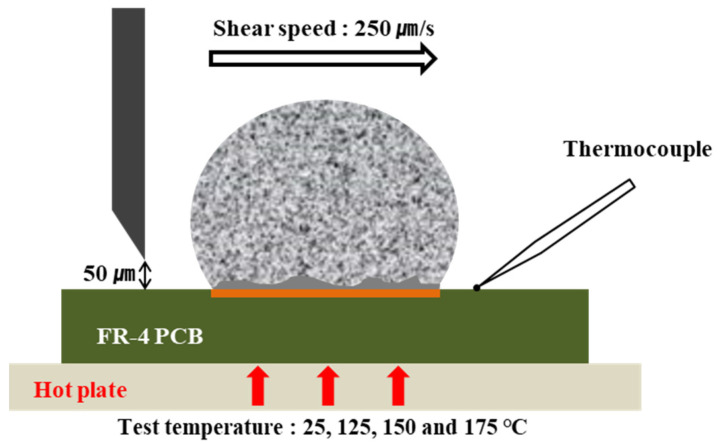
Schematic diagram and test conditions for the shear tests.

The hardness of the solder was measured with a Vickers hardness tester (HM-220A). A load of 300 g was applied and held for 10 s. To measure the hardness, 1 cm^3^ of each solder was prepared and the measurement surface was polished. The average value was calculated by taking 10 measurements for each solder. A bonding strength tester (DAGE-BT4000) that was equipped with a 5 kg load cell was used to evaluate the mechanical properties according to the number of reflows. In this experiment, the test was conducted on a hot plate set to 25, 125, 150, and 175 °C to observe changes in the bonding strength as a function of the temperature. The shear height was 50 µm and the shear speed was 250 µm/s, and 22 shear strengths were measured and averaged per solder composition. Figure 4 shows a schematic of the shear strength measurement and measurement conditions.

## 3. Results

**Figure 5:**
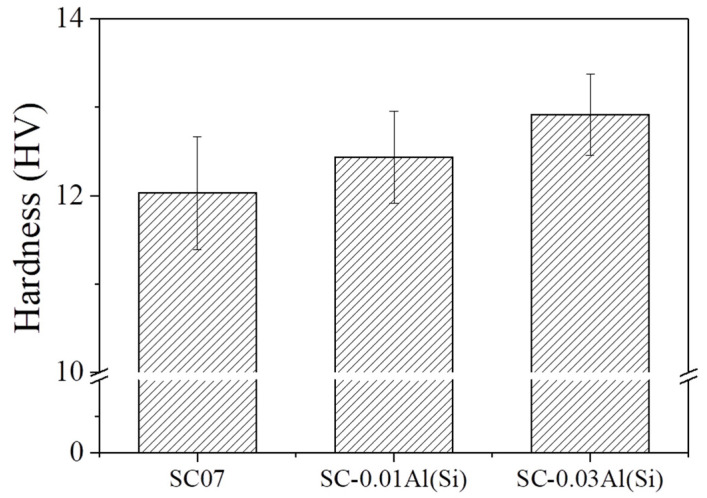
Hardness of the SC07, SC-0.01Al(Si), and SC-0.03Al(Si) solders [27].

Figure 5 shows the hardness of the SC07, SC-0.01Al(Si), and SC-0.03Al(Si) solders after the as-reflow [27]. The hardnesses of the SC-0.01Al(Si) and SC-0.03Al(Si) solders were approximately 4% and 7% higher than those of the SC07 solder. This appears to be because the Al and Al-Cu IMCs dispersed inside the solders blocked the propagation of cracks that occurred during the hardness test [28].

**Figure 6:**
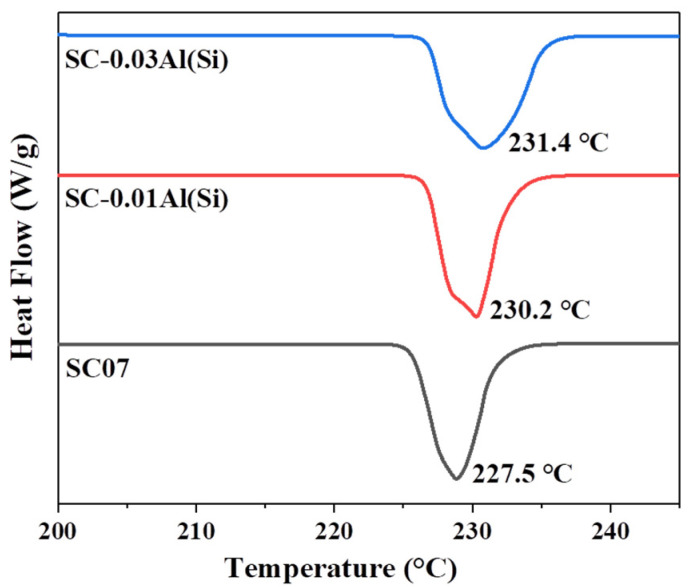
DSC results for the SC07, SC-0.01Al(Si), and SC-0.03Al(Si) solders.

Figure 6 shows the results of measuring the melting points of the three solders using DSC. The melting points were measured to be approximately 227.5 °C for the SC07 solder, 230.2 °C for SC-0.01Al(Si) solder, and 231.4 °C for the SC-0.03Al(Si) solder. Considering that the process temperature of the SC07 solder alloy was 227 °C, the melting point of SC-0.01Al(Si) was approximately 3 °C higher, and that of SC-0.03Al(Si) was 4 °C higher. The addition of Al(Si) appears to have slightly increased the melting points of the solders through reduced supercooling [29,30].

**Figure 7:**
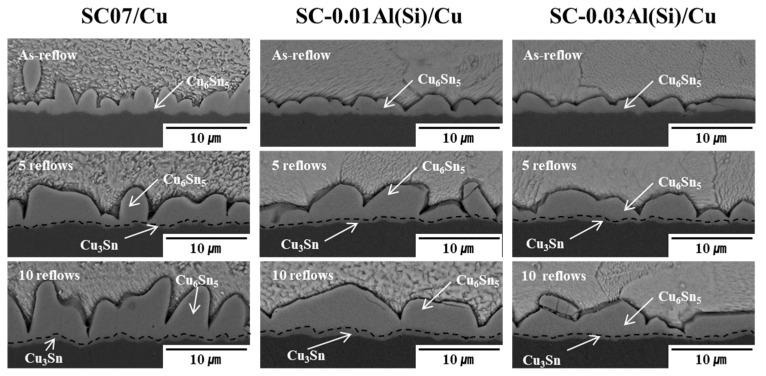
SEM images showing the interfacial IMCs of the solder joints after the as-reflow, 5 reflows, and 10 reflows.

Figure 7 shows the SEM images of the solder joints of each solder after multiple reflows. In the as-reflow specimen of the SC07 solder, the total IMCs grew significantly compared with the SC-0.01Al(Si) and SC-0.03Al(Si) solders, and no Cu_3_Sn was observed using SEM. Elongated, scallop-shaped Cu_6_Sn_5_ IMCs were observed in the SC07 solder, and round, scallop-shaped Cu_6_Sn_5_ IMCs were observed as the Al(Si) content increased. After 10 reflows, the total IMCs of the Sn-0.7Cu solder had grown the most significantly. This appears to have occurred because the Al compounds present near the grain boundary or interface inhibited the formation and growth of interfacial IMCs by blocking the diffusion of Sn and Cu atoms [31,32]. Cu_3_Sn IMCs were similarly observed in all three solders.

**Figure 8:**
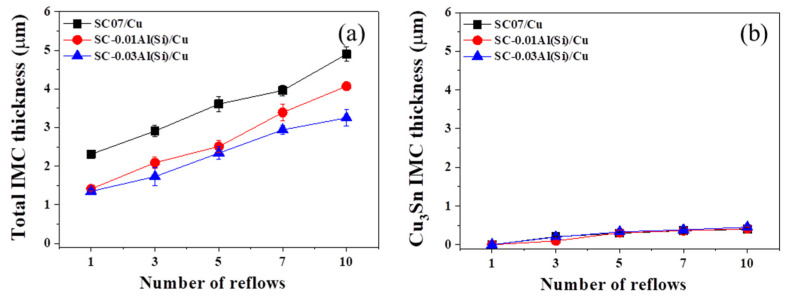
Plots of the IMC thickness versus the number of reflows: (**a**) total IMC thicknesses and (**b**) Cu_3_Sn IMC thicknesses.

Figure 8 shows the IMC thicknesses of the SC07, SC-0.01Al(Si), and SC-0.03Al(Si) solders as a function of the number of reflows. The total IMC thicknesses at the beginning were found to be 2.8 µm for the SC07 solder, ≈1.79 µm for the Sn-0.5Cu-0.01Al(Si) solder, and ≈1.52 µm for the Sn-0.5Cu-0.03Al(Si) solder. The total IMC thicknesses of the SC-0.01Al(Si) and SC-0.03Al(Si) solders were approximately 36% and 46% lower, respectively, than those of the SC07 solder. The IMC thicknesses increased as the number of reflows increased for all three solders. After 10 reflows, the total IMC thicknesses were found to be 4.7 µm for the SC07 solder, ≈4.02 µm for SC-0.01Al(Si), and ≈3.25 µm for SC-0.03Al(Si). In addition, the Cu_3_Sn IMCs increased as the number of reflows increased, but there was no significant difference according to the Al(Si) content. Cu_6_Sn_5_ IMCs are grown by the mutual diffusion and reaction of Cu/Sn, whereas Cu_3_Sn IMCs are grown via reactions in Cu/Cu_6_Sn_5_ IMCs [33]. It appears that the addition of Al(Si) interfered with the mutual diffusion of Cu/Sn, but it did not affect the reaction of Cu_3_Sn that occurs in Cu/ Cu_6_Sn_5_ IMCs.

**Figure 9:**
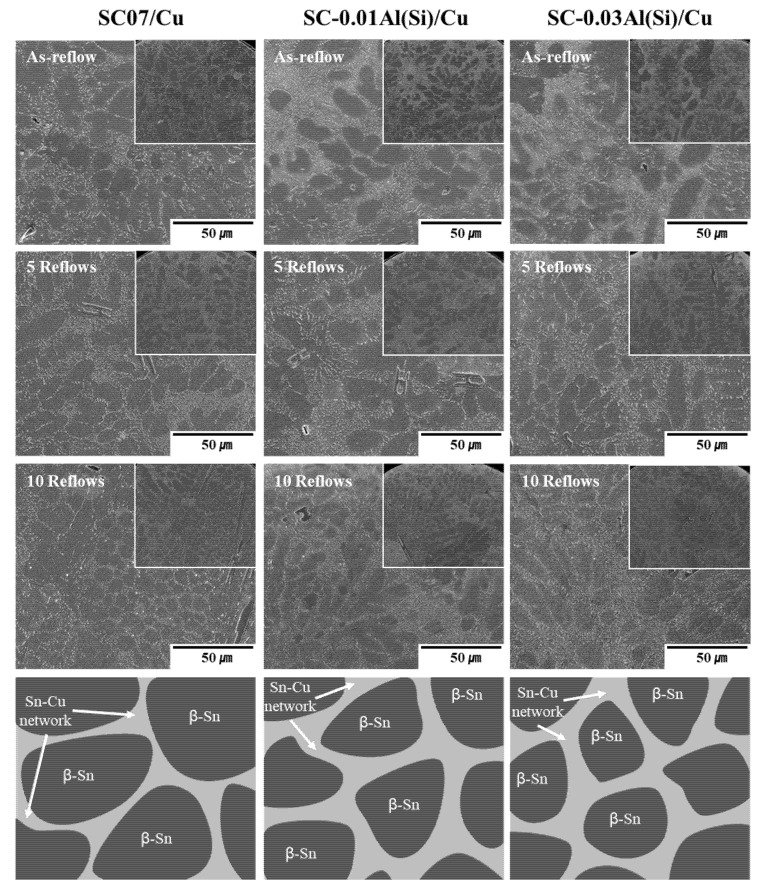
Secondary electron SEM images showing the microstructures of the solder balls under various reflows.

Figure 9 shows the microstructures of the SC07, SC-0.01Al(Si), and SC-0.03Al(Si) solders as a function of the number of reflows. The inside of the solders was composed of β-Sn dendrites, eutectic areas, and some large precipitated IMCs [34]. After the as-reflow, the inside of the solders was composed of β-Sn areas surrounded by eutectic areas containing Cu_6_Sn_5_ IMCs, and β-Sn areas surrounded by refined eutectic areas were observed as the Al(Si) content increased. Even after 10 reflows, the Cu_6_Sn_5_ IMCs were more finely distributed inside the solders as the Al(Si) content increased, indicating that Al(Si) inhibited the coarsening of the Cu_6_Sn_5_ IMCs. Even a minute amount of Al(Si) effectively facilitated the formation of the refined β-Sn + Cu_6_Sn_5_ network. No Al(Si) was observed using SEM because its content level was quite low. Therefore, the Al(Si) inside the solders was observed using TEM and EPMA.

**Figure 10:**
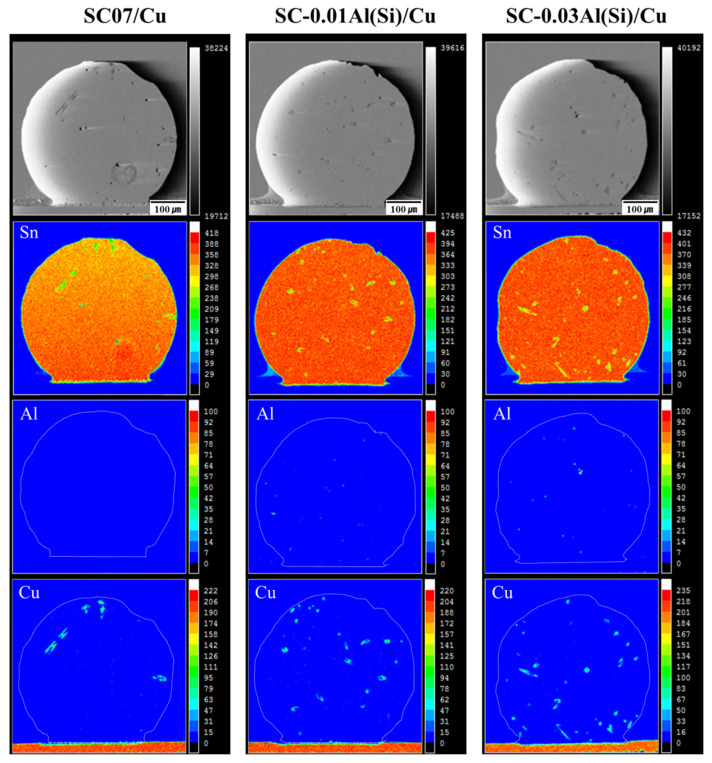
EPMA electron mapping of various solder joints after 10 reflows [27].

Figure 10 shows the observations of the inside of the solders using EPMA after 10 reflows. The Cu_6_Sn_5_ IMCs of the SC07 solder were coarser than those of the SC-0.01Al(Si) and SC-0.03Al(Si) solders. In addition, finely distributed Cu_6_Sn_5_ IMCs were observed inside the solders. Al or Al IMCs were observed inside the solders containing Al(Si) and their amounts increased as the Al(Si) content increased. According to Koo et al., it was found that when Al was added to Sn-0.7Cu, the solidification temperature increased [35]. In the liquid phase, the Al or Al IMC phase was first formed and served as a nucleation site during coagulation. As a result, the particle size of the β-Sn was refined.

**Figure 11:**
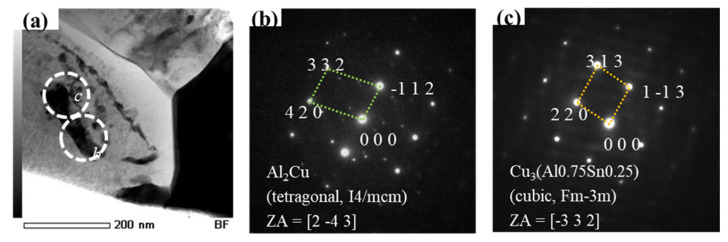
(**a**) TEM bright-field image of the interfacial microstructure for the SC-0.03Al(Si) solder joint. Selected area electron diffraction patterns for (**b)** Al_2_Cu and (**c**) Cu_3_(Al_0.75_Sn_0.25_) [27].

Thermodynamic calculations and experiments have shown that the ternary Sn-Al-Cu system has a large stable liquid miscibility gap [36,37], through which Al-Cu IMCs should traverse through Al-added solder alloys during soldering. According to a report by Kotadia et al., when Al was added to the SAC305 solder, Al_2_Cu IMCs were observed after the reflow [38]. Figure 11 shows the TEM image of the SC-0.03Al(Si) solder and the selected area electron diffraction (SAED) patterns after 10 reflows. In Figure 11b,c, Al-Cu IMCs and Cu-Al-Sn IMCs were observed. The SAED patterns are consistent with the reported crystal structures of Al_2_Cu IMCs and Cu_3_(Al_0.75_Sn_0.25_) IMCs [35,38].

**Figure 12:**
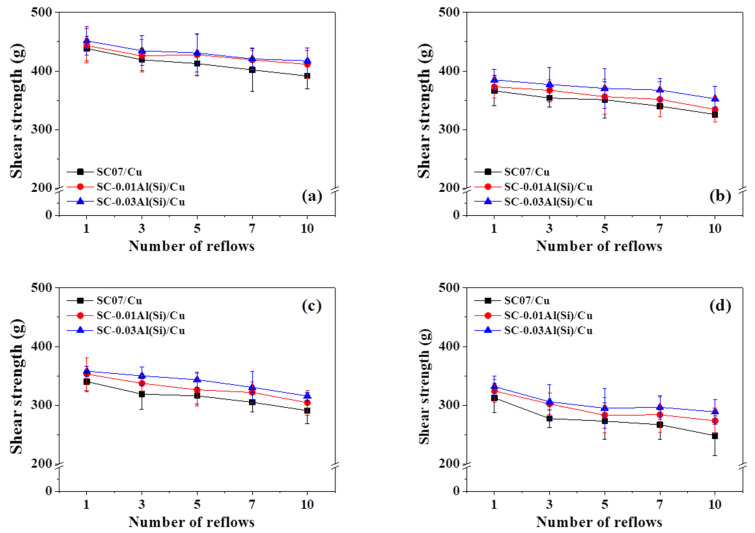
Shear strength of solder joints under various reflows at (**a**) 25 °C, (**b**) 125 °C, (**c**) 150 °C, and (**d**) 175 °C.

Figure 12 shows the shear strength according to the shear temperature and the number of reflows. For all the solders, the shear strength decreased as the shear temperature increased. It has been generally reported that tensile strength decreases and the ductility of an alloy increases as the temperature increases [39,40]. Under all conditions, the solders containing Al(Si) exhibited higher shear strength than the SC07 solder, and the reduction in shear strength increased as the number of reflows increased. The addition of Al(Si) appears to have increased the shear strength owing to the finer β-Sn particles. According to the Hall–Petch relationship, fine β-Sn particles exhibit higher strength [41]. In addition, the shear strength of the SC-0.03Al(Si) solder was slightly higher than that of the SC-0.01Al(Si) solder. This appears to be because the Al_2_Cu IMCs or Cu_3_(Al_0.75_Sn_0.25_) IMCs that were formed in larger quantities in the SC-0.03Al(Si) solder acted as new reinforcing particles and interfered with dislocations in a manner similar to that of Cu_6_Sn_5_ IMCs.

**Figure 13:**
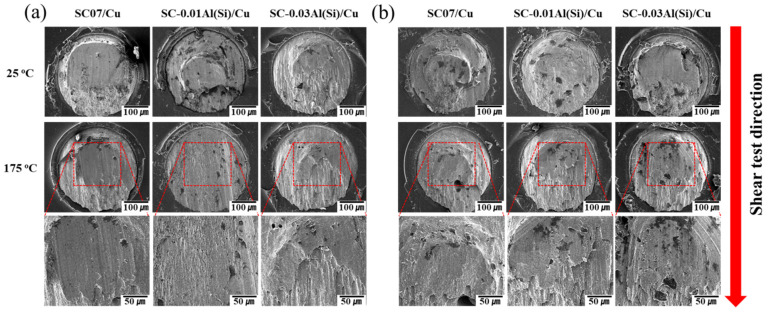
Fracture surfaces of the solder joints under various shear speeds: (**a**) as-reflow and (**b**) 10 reflows.

Figure 13 shows the fracture surfaces of the solder joints during the shear test. In general, fracture occurs at the interface or in the solder region with the lowest strength in the solder ball shear test [42]. In addition, the mechanical properties of the solder joint may be significantly degraded if IMCs with excessive thicknesses are formed between the solder and the substrate [43]. In this study, however, cracks always occurred inside the solders, regardless of the shear temperature or the number of reflows. In addition, the elongation of the inside of the solders increased as the shear temperature increased.

## 4. Conclusions

In this study, the microstructural changes and mechanical properties of SC07, SC-0.01Al(Si), and SC-0.03Al(Si) solders were investigated according to the number of reflows. The results of this study can be summarized as follows:

1. In the DSC analysis, the melting points were measured to be approximately 227.5 °C for the SC07 solder, 230.2 °C for the SC-0.01Al(Si) solder, and 231.4 °C for SC-0.03Al(Si) solder. The addition of Al(Si) appears to have slightly increased the melting points of the solders through reduced supercooling.
2. The cross-sectional analysis revealed that the IMCs of the SC-0.03Al(Si) solder grew less under all reflow conditions. No Cu_3_Sn IMCs were observed at the beginning, but they were grown and attained similar thicknesses in all three solders after 10 reflows.
3. When the inside of the solders was analyzed, it was found that the IMCs were the most finely dispersed inside the SC-0.03Al(Si) solder, and they were also the most finely dispersed inside the solder after 10 reflows.
4. The EPMA and TEM analysis results showed that Al-Cu IMCs and Cu-Al-Sn IMCs were observed inside the solders containing Al and Si, and their amounts increased as the Al content increased.
5. The highest shear strength was obtained for the SC-0.03Al(Si) solder. For all solders, the shear strength decreased with increasing shear temperature. When the fracture surfaces were analyzed, ductiles fracture was observed in all three solders. The addition of Al appears to have increased the solder strength by finely distributing the IMCs inside the solder.

## Figures and Tables

**Table 1:** Composition of the manufactured solder alloys.

Solder Alloy	Sn (wt.%)	Cu (wt.%)	Al (wt.%)	Si (wt.%)
SC-0.01Al(Si)	Bal.	0.6527	0.0140	0.0014
SC-0.03Al(Si)	Bal.	0.6620	0.0307	0.0030

## Data Availability

Data sharing is not applicable to this article.
